# ImaginYouth—A Therapist-Guided Internet-Based Cognitive-Behavioral Program for Adolescents and Young Adults With Body Dysmorphic Disorder: Study Protocol for a Two-Arm Randomized Controlled Trial

**DOI:** 10.3389/fpsyt.2021.682965

**Published:** 2021-05-25

**Authors:** Andrea S. Hartmann, Michaela Schmidt, Thomas Staufenbiel, David D. Ebert, Alexandra Martin, Katrin Schoenenberg

**Affiliations:** ^1^Institute of Psychology, Clinical Psychology and Psychotherapy, Osnabrück University, Osnabrück, Germany; ^2^Institute of Psychology, Research Methods, Diagnostics and Evaluation, Osnabrück University, Osnabrück, Germany; ^3^Department of Sport and Health Sciences, Psychology and Digital Mental Health Care, Technical University Munich, Munich, Germany; ^4^School of Human and Social Sciences, Clinical Psychology and Psychotherapy, University of Wuppertal, Wuppertal, Germany

**Keywords:** adolescents, cognitive behavioral therapy, internet-based intervention, appearance concerns, body dysmorphic disorder, young adults, e-mental health

## Abstract

**Background:** Body dysmorphic disorder (BDD) is a relatively common mental disorder in adolescents and young adults, and is characterized by severe negative psychosocial consequences and high comorbidity as well as high mortality rates, mainly due to suicides. While patients in Germany have health insurance-financed access to evidence-based outpatient treatments, that is, cognitive-behavioral treatment (CBT), waiting lists are long. Furthermore, patients with BDD report diverse treatment barriers, primarily feelings of shame and the belief that they would be better off with treatments that would alter the perceived flaw(s). Given adolescents' and young adults' high affinity to electronic media, the accessibility of evidence-based care for this severe mental disorder could be improved by providing an internet-based therapist-guided CBT intervention.

**Methods:** In a two-arm randomized controlled trial (*N* = 40), adolescents and young adults (15–21 years) with a primary diagnosis of BDD based on a semi-structured clinical expert interview will be randomly allocated to an internet-based therapist-guided CBT intervention or a supportive internet-based therapy intervention. Assessments will take place at baseline, after mid-intervention (after 6 weeks), post-intervention, and at 4-week follow-up. The primary outcome is expert-rated BDD symptom severity at the primary endpoint post-intervention. Secondary outcomes include responder and remission rates based on expert rating, self-reported BDD symptoms, and psychosocial variables associated with BDD.

**Interventions:** The CBT-based intervention consists of six modules each comprising one to three sessions, which focus on psychoeducation, cognitive restructuring, work on self-esteem, exposure and ritual prevention, mirror retraining, and relapse prevention. A study therapist provides feedback after each session. The supportive therapy intervention consists of access to psychoeducational materials for the same 12-week period and at least one weekly supportive interaction with the study therapist.

**Conclusions:** This is the first study to examine the feasibility and efficacy of an internet-based therapist-guided CBT intervention in adolescents and young adults with BDD. It could be an important first step to increase accessibility of care in this age group and for this severe and debilitating mental disorder.

**Clinical Trial Registration:** German Register of Clinical Studies, DRKS00022055.

## Introduction

Body dysmorphic disorder (BDD) is characterized by one or more perceived flaws in one's body, which are not visible or appear minor to others [Diagnostic and Statistical Manual of Mental Disorders, DSM-5; (1)]. Appearance-related concerns can be delusional [i.e., individuals do not understand that their perception of their own appearance differs from reality; (2)], and there is evidence that BDD is linked to more delusional concerns in adolescence than in adulthood (3). These appearance-related concerns lead to negative emotions such as shame, anxiety, and depression (4). Moreover, they are accompanied by so-called safety behaviors, which are compulsive and mental rituals performed in order to check, fix, or improve the perceived flaw(s) (1). Further typical examples of safety behaviors include rumination processes (5) and avoidance of situations in which one is confronted with one's appearance flaws or in which the perceived flaws are exposed to others [e.g., looking at oneself in the mirror, going to a party; (6)].

Various studies hint at a similar phenomenological picture of BDD in adolescents and adults [e.g., (3, 7–14)]. BDD is highly debilitating, with case studies in children and adolescents showing that BDD sufferers achieve poorer school grades (15), give up leisure time activities (12), miss a lot of school (16) or even drop out of school (15), withdraw socially (12), and might even become housebound (14). Comorbidity is the rule rather than the exception (14), and the disorder is characterized by low self-esteem and low quality of life [e.g., (17, 18)] as well as high rates of suicidality (19). Thus, BDD is a severe mental disorder, for which adolescence is a critical age period given the mean age of onset at 16 years (20) and the particularly high prevalence rate of 3.6% among 15–21-year-olds (19). Against this background, the need for adequate treatment for this age group is clear.

Two meta-analyses summarized the growing number of randomized controlled treatment trials in BDD, and concluded that cognitive-behavioral therapy and a pharmacological intervention with selective serotonin reuptake inhibitors (SSRIs) represent first-choice treatments (21, 22). Evidence in adolescents is far more limited, although case studies also support the effectiveness of CBT (15, 23–25) and SSRIs (14–16, 24, 26). Two larger studies established the feasibility of CBT for BDD in adolescents (26) and the superiority of CBT over a control condition consisting of telephone monitoring and psychoeducational materials (27).

Despite being a therapy method that is financed by health care insurers, waiting lists for CBT for adolescents in Germany are long (28). Furthermore, BDD might still be relatively unknown to practitioners and appearance concerns might be mistaken for age-appropriate concerns that do not require therapeutic attention. Additionally, many patients with BDD are very reluctant to seek psychological treatment (29), mainly due to shame, but also the idea that a treatment that changes the perceived flaw (i.e., plastic surgery, dermatology) might be more suitable for their problem. Given the highly relevant role of body image in the transitions occurring during adolescence (30) and the long-term severe consequences for future development potentially caused by BDD, an adequate treatment of BDD in adolescence is indispensable but is hindered, among other things, by the aforementioned factors.

One possibility to overcome these hindrances is an internet-based intervention. On the one hand, adolescents show a high affinity to electronic media and high acceptance rates of internet-based interventions (31). On the other hand, internet-based interventions have proven to be effective for various mental disorders in children and adolescents [for a meta-analytic overview: (32)]. The applicability of this modality for BDD treatment is further supported by a study which demonstrated the effectiveness of an internet-based therapist-guided CBT intervention in adults with BDD (33). Therefore, we developed a therapist-guided internet-based cognitive-behavioral (CBT) intervention in the style of an adult therapist-guided internet-based CBT intervention, which focused on clinical and subthreshold forms of BDD (Schoenenberg and Martin, unpublished manuscript: Online Behandlung der Körperdysmorphen Störung: Das imagin Programm [Online treatment of Body Dysmorphic Disorder: the “Imagin” program]; DRKS00015626) and was inspired by CBT manuals for the treatment of BDD (34, 35).

## Aims of the Study

The main aim of this study is to evaluate the efficacy of an internet-based therapist-guided CBT intervention (ImaginYouth) for reducing symptoms of BDD in adolescents and young adults (in the following named adolescents) and to evaluate its superiority over a supportive online therapy condition (SOT) from pre- to post-intervention. Furthermore, we aim to assess the stability of both efficacy and superiority to follow-up, and the acceptability and feasibility of the intervention format.

We hypothesize that the ImaginYouth intervention will reduce primary expert-rated BDD symptom severity from pre- to post-intervention. Further, we expect the reduction of primary BDD symptom severity to be greater in the ImaginYouth than in the SOT intervention group from pre- to post-intervention. Secondary analyses include the testing of the above-mentioned hypotheses regarding self-reported BDD-symptom severity and associated (comorbid) symptoms. Additionally, we hypothesize that the number of remitted patients and responders will be higher at post-intervention in the ImaginYouth than in the SOT intervention group. Moreover, we expect both the changes and differences between the conditions at post-intervention to be stable over the 4-week follow-up period, and thus hypothesize a significant symptom reduction from pre-intervention to follow-up and a larger symptom reduction from pre-intervention to follow-up in the ImaginYouth compared to the SOT intervention.

## Methods

### Design

**Figure 1:**
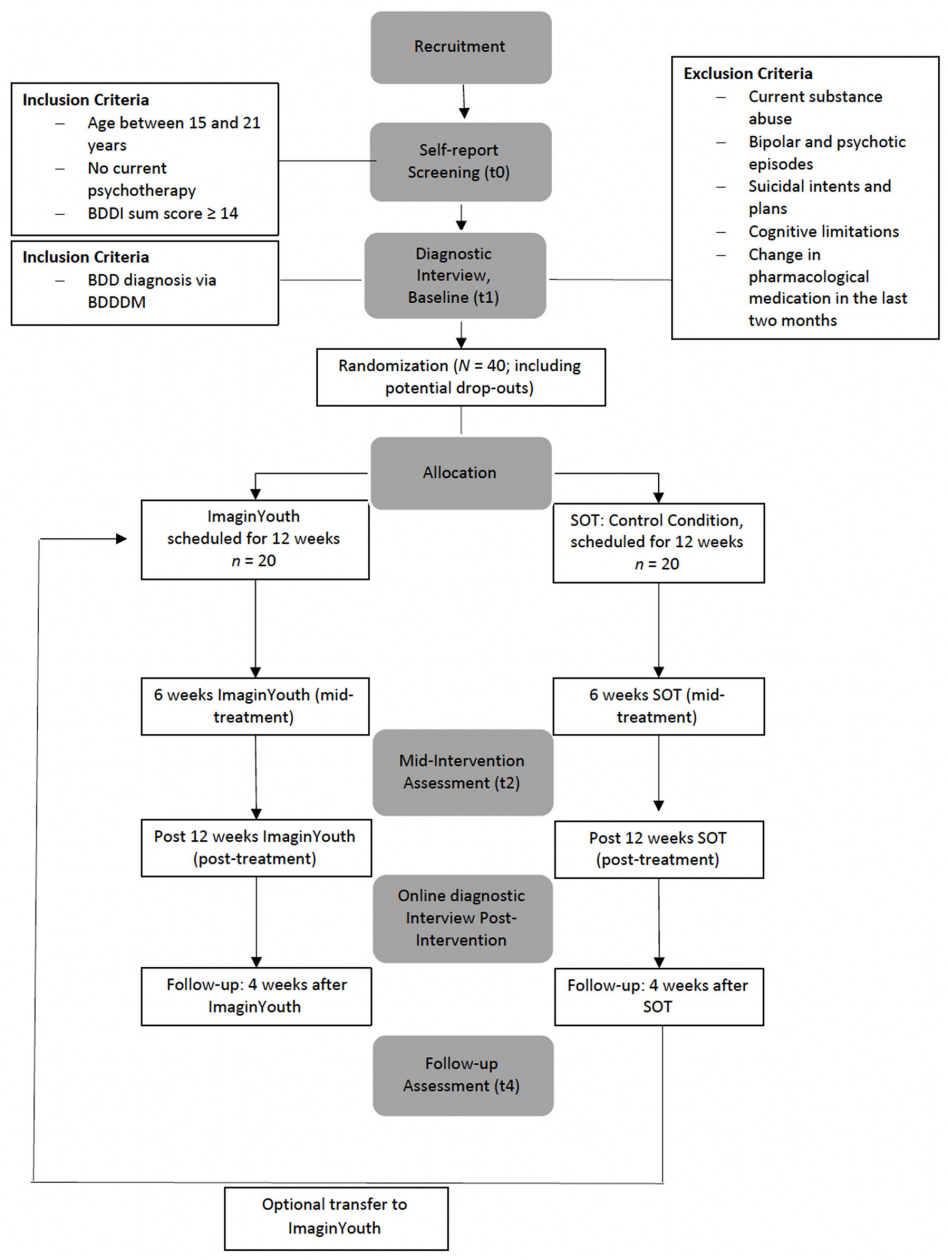
Flow chart illustrating the study design.

A two-arm randomized controlled trial (RCT) will be conducted to evaluate the internet-based therapist- guided CBT intervention compared to a supportive online therapy intervention. After screening (t0), assessments will take place at baseline (t1), mid-intervention (t2), post-intervention (t3), and 4-week follow-up (t4). Figure 1 provides a detailed overview of the study design. The mid-intervention assessment will not be a focus of the main study and therefore of this protocol, but will be used to identify, for example, predictors and early responders in secondary exploratory analyses. All procedures involved in the study will be consistent with the generally accepted standards of ethical practice approved by the Osnabrück University ethics committee (Ethik-35/2018). The trial is registered in the German Register of Clinical Studies (Deutsches Register Klinischer Studien; DRKS00022055).

### Participants and Procedures

#### Inclusion and Exclusion Criteria

Inclusion criteria include age between 15 and 21 years, a primary diagnosis of BDD (comorbid diagnoses are allowed) established by the expert interview Body Dysmorphic Disorder Diagnostic Module for DSM-5 [BDDDM; (36)] after a previous sum score of ≥14 on the Body Dysmorphic Symptoms Inventory [BDDI as is in the Table; (37)] during the screening. Exclusion criteria current substance abuse, bipolar or psychotic episodes, suicidal intent and plans, current psychotherapy, cognitive limitations that would hinder work on the intervention, and/or a change in psychopharmacological medication in the last 2 months.

#### Recruitment

Recruitment will take place in mainly German-language countries (i.e., Germany, Austria, Switzerland) and will mostly be performed online through social media (e. g., Instagram, Facebook, YouTube), the study's website, and thematic websites. Furthermore, we will announce the start of the study (including in-depth information on the intervention and contact details) in a University press release, in newspapers, youth-specific magazines, student mailing lists/newsletters, student counseling centers or organizations, and to therapists affiliated with Osnabrück University.

#### Assessment of Eligibility and Randomization

After participants have contacted us by telephone, email or via our social media channels, they will be provided with a link to the online self-report screening (t0, in Unipark; EFS Survey, Questback GmbH, Cologne) through a self-chosen email address. If they fulfill the first inclusion criteria (age, BDDI as is in Table sum score ≥14, and no current psychotherapy), the diagnostician (a clinical psychologist in training) will contact them by email in order to make an appointment to (1) conduct the informed consent procedure and (2) complete the structured clinical interviews. The diagnostician is blind to the condition of the participant both at pre- and post-assessment. In case of crossover (i.e., a participation in ImaginYouth after having participated in SOT before) the ratings will not be blind anymore. The diagnostician will also provide the participants with detailed written information on the study procedure, our privacy policy and a note that it is possible to withdraw from the intervention at any time without facing any consequences. The informed consent procedure and the interviews will be conducted online using RedConnect (RED Medical Systems GmbH, Munich), the latter only after the informed consent form has been sent to the diagnostician by email. If the participants fulfill the remaining inclusion and exclusion criteria, they will then be randomized to one of the two intervention conditions by our external collaboration partners (AM and KS) who, from this point, will not be further involved in the study. A full randomization of the prospective 40 cases will be generated via a list randomizer (RANDOM.ORG, Randomness and Integrity Services Ltd, Dublin) by KS. Once randomization has been conducted, the second first-author (MS; study therapist in both conditions; clinical psychologist in training) will provide participants with a log-in for the intervention to which they have been allocated on the e-mental-health platform Minddistrict (Minddistrict, Berlin).

### Intervention

#### ImaginYouth Condition

The development of the therapist-guided internet-based CBT intervention was inspired by a therapist-guided internet-based CBT intervention for adults with clinical and subthreshold BDD, “the Imagin program” (Schoenenberg and Martin, unpublished manuscript). From this program, we adopted the overall structure as well as some exercises, headers, images and videos. However, this was strongly adapted to fit the younger participants. As such, we introduced a different order of modules, a different emphasis on certain topics over others, mostly new psychoeducational reading materials, exercises and homework, a different underlying etiological model, and a different structure and composition of single sessions.

ImaginYouth consists of six modules comprising one to three sessions each. Each session can be completed in about 30–60 min and participants are advised to find a specific day on which they can take part in one session on the same day each week. If participants stick to their schedule, the intervention will last for 12 weeks. At the beginning of each session, participants are asked to report on their experiences with the last session and the homework in order to give them the opportunity to reflect on their BDD development. Furthermore, they have the chance to report on their current overall emotional state and to answer an open question on this week's particular life events. Sessions consist of psychoeducational materials on the topic being worked on (e.g., safety behaviors), including summarizing recap paragraphs and optional drop-down “Read more” sections to facilitate reading. This focus is due particularly to the diverse nature of the sample, especially in terms of age and cognitive abilities. Moreover, we included optional drop-down “Practical tips” sections for some of the exercises to provide further support if needed, as well as optional drop-down “Fun fact!” sections to enhance the overall experience of undergoing the session. Overall, attempts were made to tailor the sessions to the individual needs of the participants and to give the young participants a sense of autonomy and co-determination. The written content was enhanced by drawings that emphasize the information given as well as video and audio clips. Furthermore, participants are asked to complete mandatory exercises to apply the material to their own behavior and/or perception. The content and the exercises are accompanied by three teenage case descriptions (two girls, one boy) that are introduced in the first session. These serve as relatable examples by sharing their experiences with and everyday-life application of the current topic of the session and/or sharing their answers in the exercises to give participants a better idea of what they are being asked to do. Another connecting element of the sessions is the BDD model that is introduced in the second session. This model links to the current topic at the beginning of each session to explain the rationale for the proposed exercises, and aims to increase participants' motivation. At the end of each session, participants are asked to complete a short survey (see section Assessment and Data Management). Between sessions, participants work on homework that aims to facilitate transfer to the real-world setting (e.g., diaries, exposure exercises); this is often enhanced by “cheat sheets” summarizing the crucial content of the previous session. After each session, participants receive written feedback from the study therapist on the exercises and homework through the program's chat function. At the end of each module, participants can download a handout summarizing the crucial aspects of the module in order to create their personal BDD “toolbox.” After the completion of each session, further content is automatically released. New modules need to be released by the study therapist. While the sessions and the integrated chat can only be accessed *via* a web browser, the homework is also available on the complementary smartphone app. Detailed information on the number of sessions per module and content are illustrated in Table 1.

**Table 1:** Description of modules, their number of sessions and content, and associated homework.

**Module**	**Number of sessions**	**Content**	**Homework**
Psychoeducation	2	Introduction to symptoms, prevalence and multifactorial development of BDD as well as therapy options; Introduction to the three accompanying case studies Bilal, Frieda and Birte	–
		Introduction to the BDD-model “The Vicious Circle of BDD”: development and maintenance of BDD; Optional information sheet on BDD for relatives and friends	Diary: Monitoring symptoms of BDD and time spend on preoccupation with flaw(s)
Automatic thoughts	2	Introduction to the link between thoughts and emotions; Identification of automatic dysfunctional thought patterns and errors in reasoning	Diary: Monitoring errors in judgement and associated feelings
		Questioning and replacing dysfunctional thoughts with more realistic, functional thoughts	Diary: Detecting, questioning and replacing errors in judgement with functional thoughts
Self-depreciating thoughts	1	Identification and remodeling of specific self-depreciating thoughts (“My inner critic”); Identification of positive character traits and values to enhance self-esteem	Interviewing three people on one's own positive character traits
Safety behavior	3	Problematization, identification und reduction of compulsive appearance-related rituals	Monitoring and reduction of two rituals
		Problematization and identification of avoidance behavior; Introduction to exposure therapy	Setting up an exposure plan; Exposure to one avoided situation
		Exposure therapy, continued: detecting typical obstacles and how to overcome them	Setting up an exposure plan; Exposure to three avoided situations
View in the mirror	3	Consequences of avoided or compulsive mirror checking; Associated selective attention processes and cognitive errors	Diary: Mirror checking protocol
		Introduction to a more functional view in the mirror and mirror exposure	Mirror exposure with pre-recorded audio instructions
		Mirror exposure, continued: refraining from safety behavior	Three mirror exposures with pre-recorded audio instructions
Future path/relapse prevention	1	Planning of further recovery steps; Relapse prevention: How to handle stressful events	-

#### Supportive Online Therapy Condition

A supportive therapy condition was chosen as the control condition, as this has proven to be feasible in BDD in adults (33) and in children and adolescents with depression or anxiety symptoms (38, 39). In the SOT condition, patients can read psychoeducational material on BDD for 12 weeks (symptoms, prevalence, development, maintenance, therapy options, case examples) that is identical to the psychoeducational reading material from the first module of the ImaginYouth condition, but without exercises to facilitate transfer to the patient's individual case or the identification of intervention targets. Through the program's messaging function, the control patients are contacted weekly (repeated in case of non-responding) by the study therapist. In this communication, the therapist asks about their current well-being as well as their experiences, thoughts, and emotions regarding BDD symptoms and how these influence participants' lives if participants do not bring up their own questions or concerns.

### Safety Management

Given the high suicidality in BDD (19), we aim to provide a thorough safety management in our study. First, potential participants with concrete suicidal intentions and plans identified during screening and/or diagnostic sessions will be excluded from participation and will be informed about potential places to seek treatment in their area of residence. During the intervention, we will have a weekly monitoring of suicidal ideation in place, which the study therapist has to consult prior to interaction with the patient. If suicidal ideation develops, a stepped-care plan will be set in motion, including anti-suicide contracts, a telephone call with the study therapist in which a stepped emergency plan is developed, a telephone call with the supervisor (AH; a clinical psychologist with expertise in treatment of BDD), and involvement of treatment providers in the patient's area of residence. In order to track the general development of all patients, a fortnightly supervision session will be held. The supervisor will be accessible for the study therapist during the whole project. For *post-hoc* analyses, we will also assess adverse events during and after the intervention (see measures) to assess its moderation of treatment effects.

### Quality Management

Quality management (i.e., adherence to condition and engagement) will include the aforementioned fortnightly supervision session, measurement of the time used to communicate with each patient as well as number of messages. Messages between patients and study therapist are in written format only, are recorded, and can be subject to content analysis of adherence to condition during data analysis. Furthermore, diagnostic sessions will be recorded and interrater reliability will be calculated in 20% of randomly chosen cases. The diagnostician will be blind to the randomization of the participants and will be replaced if deblinded.

### Assessment and Data Management

Detailed self-reports will take place at screening (t0), baseline (t1) prior to randomization, mid-intervention (t2), post-intervention (t3), and 4-week follow-up (t4; see Figure 1 for a detailed overview). On a more regular basis, we will gather brief self-reports on suicidal intent and negative emotions (after each session) as well as on BDD and depressive symptoms (after each module). Self-reported data (t0 through t4) will be collected using the secure online-based assessment system Unipark (EFS Survey, Questback GmbH, Cologne). Expert interviews will be conducted over the internet at t1 and t3 *via* the secure online video conferencing platform RedConnect (RED Medical Systems GmbH, Munich).

### Outcomes

#### Primary Outcomes

*Yale-Brown Obsessive-Compulsive Scale Modified for Body Dysmorphic Disorder* [BDD-YBOCS; (40); German version: (41)]. The main primary outcome assessing severity of BDD symptoms will be the semi-structured expert clinical interview BDD-YBOCS (40). This will also be used to assess responder and remission status (42). The BDD-YBOCS is the gold standard instrument and assesses both cognitive (e.g., time occupied by thoughts about body defect) and behavioral symptoms (e.g., degree of control over compulsive behavior) of BDD and delusionality using a total of 12 items. The item regarding delusionality will be rated again within the participants' answers on the Brown Assessment of Beliefs Scale [BABS; (43)]. Items on the BDD-YBOCS are rated on a 5-point Likert Scale from 0 to 4, with the scale content depending on the respective item (e.g., 0 = *none* to 4 = *extreme [spends more than 8 h/day on these activities]*). The scale has shown good internal consistency (0.80) and interrater reliability [0.99; (40)].

#### Secondary Outcomes

Detailed information on number of scales, items, content scaling, and psychometric properties of all secondary measures (both for further BDD-symptoms and comorbid psychopathology) included in this protocol and primary analyses are highlighted in light gray in Table 2. Table 2 also presents all other variables assessed in the study but not in the focus of the main analyses of the study. Some measures will be administered after each session and/or each module, which is indicated by superscripted post-module or post-session mention.

**Table 2:** Description of instruments and psychometric characteristics as well as points of measurement.

**Instrument**	**Author (German-language version)**	**Construct**	**Number total items**	**Subscales**	**Scale**	**Cron bach‘s α**	**Time point**				
							**0**	**1**	**2**	**3**	**4**
**Secondary BDD symptoms**											
Body dysmorphic disorder diagnostic module^I^	KDS-Net (unpublished manuscript)	DSM-5 based Diagnosis of BDD	8	–	3-point scale 1 (not applicable), 2 (subclinically applicable), and 3 (present/applicable)	0.96		x		x	
Brown assessment of beliefs scale^I^	(43, 44)	Delusional appearance-related thoughts	7	–	5-point Likert scale with scale content depending on the item (e.g., 0 (*absolutely sure that belief is wrong)* to 4 (*absolutely sure that belief is correct)*	0.87		x		x	
Dysmorphic concern questionnaire^Q^^,^^Post−module^	(45, 46)	Severity of BDD symptoms	7	–	4-point Likert scale from 0 (*not at all*) to 3 (*much more than other people*)	0.85	x		x	x	x
Body dysmorphic symptoms inventory^Q^	(37)	Severity of BDD symptoms	18	Specific BDD symptoms; associated characteristics	5-point Likert scale from 0 (*not at all, never, do not think about it at all*) to 4 (*very strong, more than 5 times, more than 8 h per day*)	0.88	x		x	x	x
List of safety behaviors^Q^	Martin et al. (2021)	Number and extent of safety behaviors	30	–	5-point Likert scale from 1 (*never*) to 5 (*very often*)	–		x		x	x
Mirror gazing questionnaire^Q^	Adapted from (6)	Mirror gazing behavior	8	–	Scale and scale content depending on the item (e.g., *yes*/*no*)	–		x		x	x
Physical appearance comparison scale^Q^	(47, 48)	Body-related social comparison habits	5	–	5-point Likert scale from 1 (*never*) to 5 (*always*)	0.75		x		x	x
Questionnaire of body-dysmorphic cognitions^a^^,^^Q^	Stangier and Ritter (2015); unpublished manuscript	BDD-related cognitions	20	–	5-point Likert scale from 1 (*never*) to 5 (*always*)/scale from 0 (*not convinced*) to 100 (*totally convinced*)	–	x		x	x	x
Body image questionnaire^b^^,^^Q^	(49)	Body image	20	Negative body assessment; Vital body dynamics	5-point Likert scale from 1 (*not at all true*) to 5 (*very true*)	0.84–0.91		x		x	x
**Comorbid pathology and general mental state measures**											
Diagnostic short-interview for mental disorders^c^^,^^I^	(50)	Diagnosis of comorbid symptoms (DSM-5)	–	–	Criterion fulfilled yes/partially/no	0.84–1.0		x			
Rating of negative EMOTIONS^Q^^,^^Post−session^	(51)	Arousal, anger, anxiety, sadness, disgust, stress, insecurity	7	–	5-point Likert scale from 0 (*not at all*) to 4 (*extremely*)	0.82–0.94		x		x	x
Questionnaire for social anxiety and social competence deficits for adolescents^d^^,^^Q^	(52)	Social anxiety and social competence deficiencies	44	Fear of talking and fear of being in the focus of attention; Fear of rejection; Interaction deficits; Information-processing deficits; Loneliness (additional subscale not counting for the total score)	4-point Likert scale from 0 (*never*) to 3 (*always/most of the time*)	0.76–0.90		x		x	x
Eating disorder examination questionnaire EDE-Q8	(53)	Eating disorder pathology	8	–	7-point Likert from 0 (*no day/never/not at all*) to 6 (*every day/every time/very much*)	0.93		x		x	x
Single Item Self Esteem Scale^Q^	(54, 55)	Self-esteem	1	–	7-point Likert scale from 0 (*not very true for me*) to 6 (*very true form me*)	–		x		x	x
Generalized self-efficacy scale^Q^	(56, 57)	Self-efficacy	10	–	4-point Likert scale from 0 (*not true*) to 3 (*exactly true*)	0.80–0.90		x		x	x
Multidimensional self-concept scale^Q^	(58, 59)	Estimation of self-esteem	32	Emotional self-esteem; Social self-esteem; Performance-related self-esteem; Body-related self-esteem	7-point Likert scale from 1 (*not at all/never*) to 7 (*very/always*)	0.76–0.87		x		x	x
Obsessive compulsive inventory-revised^Q^	(60, 61)	Symptoms of obsessive-compulsive disorder	18	Checking; ordering; hoarding; obsessing; washing; neutralizing	5-point Likert scale from 0 (*not at all*) to 4 (*very strong*)	0.85		x		x	x
Generic quality of life instrument for children -revised^e^^,^^Q^	(62)	Health-related life quality	24	Physical well-being, Psychological well-being; Self-esteem, Family; Friends; Education/school	5-point Likert scale from 0 (*never*) to 4 (*always*)	0.84		x		x	x
Rumination-suppression-questionnaire^f^^,^^Q^	(63)	Rumination/suppression as emotion regulation strategies	8	Rumination; Suppression	6-point Likert scale from 0 (*not at all true*) to 5 (*exactly true*)	0.50–0.83		x		x	x
Patient health questionnaire^Q^^,^^Post−module^	(64, 65)	Depressive mood	9	–	4-point Likert scale from 0 (*not at all*) to 3 (*nearly every day*)	0.86–0.89		x		x	x
Item on suicidal thoughts and plans, beck-depression-inventory-II^Q^^,^^Post−session^	(66, 67)	Suicidal thoughts and plans	1	–	4-point scale from 0 (*I don't have any thoughts of harming myself*) to 3 (*I would kill myself if I could*)	–	x	x	x	x	x
**Intervention-related measures**											
Credibility/expectancy questionnaire^Q^	(68)	Treatment expectancy and rationale credibility	6	Expectancy; Credibility	9-point Likert scale from 1 (*not at all*) to 9 (*very*) and 11-point Likert scale from 0 to 100%	0.84–0.85		x		x	x
Technology affinity questionnaire^g^^,^^Q^	(69)	Technological affinity	19	Excitement; positive consequences; negative consequences; competence	5-point Likert scale from 0 (*not at all true*) to 4 (*exactly true*)	0.73–0.86	x			x	x
Adverse events^Q^	Adapted from (70)	Adverse events due to intervention	3	−	Yes/no; 4-point Likert scale from 0 *(not at all)* to 3 *(very strongly)*	−			x	x	x
Client satisfaction questionnaire, adapted for inpatient fields of work^h^^,^^Q^	(71)	Patient satisfaction	8	−	4-point Likert scale with scale content depending on the item [e.g., 1 (*excellent*) to 4 (*bad*)]	0.87–0.93			x	x	
Message credibility scale^Q^	(72, 73)	Program and therapist credibility	3	−	7-point Likert scale from 1 (*very badly*) to 7 (*very well*)	0.87			x	x	
Working alliance inventory—short revised^Q^	(74–76)	Therapeutic alliance	12	–	5-point Likert scale from 1 (*rarely*) to 5 (*always*)	0.81–0.91			x	x	

Q *Self-report Questionnaire*;

I *Semi-structured Interview*;

*^a−h^Translated from German-language original*:

a *Fragebogen Körperdysmorpher Kognitionen*;

b *Fragebogen zum Körperbild*;

c *Diagnostisches Kurzinterview bei psychischen Störungen*;

d *Fragebogen zu sozialer Angst und sozialen Kompetenzdefiziten – Version für Jugendliche*;

e *Ruminations-Suppressions-Fragebogen*;

f *Fragebogen zur Erfassung der Technikaffinität*;

g *Fragebogen zur Lebensqualität von Kindern und Jugendlichen − Revidierte Form*;

h *Fragebogen zur Messung der Patientenzufriedenheit*;

**Post−session:** *after every completed session of the ImaginYouth condition and simultaneously in the Online supportive therapy condition/every week*;

**Post−module:** *after every completed module of the ImaginYouth condition and simultaneously in the Online supportive therapy condition; t0 = self-report screening, t1 diagnostic inteview (baseline), t2 = self-report assessment (mid-intervention), t3 = diagnostic Interview and self-report assessment (post-Intervention), self-report assessment (follow-up)*.

#### Feasibility and Acceptability Outcomes

Feasibility and acceptability will be measured using the instruments highlighted in dark gray in Table 2.

### Statistical Methods

All statistical analyses will be conducted using SPSS Statistics (IBM; Armonk, New York, USA) and R (77). Data will be mainly analyzed on an intention-to-treat basis including all randomized participants irrespective of their completion of the intervention. Additionally, study completer analyses including only participants who completed the post-intervention questionnaire battery and interviews will be conducted. Missing data will be imputed. Dropouts and completers will be compared with respect to study variables.

The main analysis focusing on efficacy of ImaginYouth will be analyzed using a repeated measures analysis of variance (rmANOVA) comparing pre- to post- intervention scores of the primary outcome. For the main analysis focusing on superiority of ImaginYouth over the SOT intervention, we will conduct a rmANOVA in the primary outcome between the two intervention conditions and changes from pre- to post-intervention. *Post-hoc t*-tests will be used to examine main effects or interactions in more detail. Cohen's d will be reported as a measure of effect size.

Secondary analyses include the examination of categorical hypotheses [e.g., for the examination of differences between ImaginYouth and SOT in responder (defined as an empirically derived cut-off point of ≥30% reduction from baseline on the BDD-YBOCS), and remission rates (operationalized as a BDD-YBOCS score ≤16 (42))] at post-intervention using Fisher's exact test. Hypotheses on efficacy and superiority regarding self-reported BDD-symptoms severity and associated (comorbid) symptoms will also be tested rmANOVAs (see above). Stability over the follow-up period and differences therein between ImaginYouth and SOT will be tested using a rmANOVA comparing pre-intervention to follow-up scores of self-reported BDD symptom severity. Finally, to examine the feasibility and acceptability of the intervention, the respective measures will be inspected descriptively.

### Sample Size Calculation

Our a priori power analysis using G^*^Power (78) focused on the main hypothesis of a greater reduction of BDD symptoms in the main outcome measure from pre- to post-intervention in the ImaginYouth than in the SOT group. Thus, we calculated the number of participants necessary to test the interaction effect in a 2 (intervention type) × 2 (repeated measures assessment point) analysis of variance.

The power analysis was based on α= 0.05 and 1-β= 0.95, and an effect size of *f* = 0.36 [yielded by a meta-analysis on effects in internet-based intervention studies in youth by (32)]. The resulting total sample size is *n* = 28. Taking into account expected dropouts [about 30% in internet-based studies; (79)], we aim to recruit 20 patients per study arm type.

## Discussion

The aim of this two-arm randomized controlled trial is to evaluate the efficacy of an internet-based therapist-guided CBT intervention (ImaginYouth) and its superiority over SOT for BDD in adolescents. Furthermore, we aim to assess the stability in terms of both efficacy and superiority over a period of 4 weeks post-intervention, as well as the acceptability and feasibility of the intervention format. It is hypothesized that ImaginYouth will reduce primary and secondary BDD symptoms from pre- to mid-, post-, and follow-up assessments. We further expect the reduction of primary and secondary BDD symptoms to be larger in the ImaginYouth group compared to the SOT group from pre- to mid-, post-, and follow-up assessment. Additionally, we hypothesize that the number of remitted patients and repsonders at post-assessment will be higher in the ImaginYouth than in the SOT intervention group.

ImaginYouth provides participants with a CBT-based intervention which makes use of techniques that are currently the first-line treatment recommended in adults (80, 81) and have shown first efficacy in youth as well (82). The ImaginYouth intervention removes some of the treatment barriers which young individuals with BDD might face. Particularly, it might not involve as many feelings of shame. Even though participation is not completely anonymous, contact with study staff (diagnostician and study therapist) do not need to involve personal face-to-face contact. We nevertheless decided in favor of a therapist-guided intervention as guidance has also been shown to increase adherence rates in internet-based interventions (83). ImaginYouth consists of interventions on cognitive, emotional, and behavioral levels, as do treatment manuals for BDD (34, 35). Thus, effects of the intervention should be perceived and measured on all levels (see secondary measures). Moreover, as previous online CBT interventions in BDD have shown (33), effects can also be measured with regard to comorbid psychopathology (e.g., a decrease in depressive symptoms as a consequence of reduced social withdrawal or cognitive restructuring of negative thoughts).

The current design has several advantages and disadvantages. We decided in favor of an older adolescent sample mainly in order to test the intervention in individuals who are able to participate in the trial independently of their caregivers and of their own volition, in order again to limit feelings of shame when having to ask caregivers for permission. Nevertheless, in the future, the intervention could be adapted for younger adolescents and children and also expanded to be suitable for caregivers. Furthermore, we chose a randomized controlled design with an active instead of a waitlist control group, which has the advantage that we will be able to closely monitor and attend to participants in the control group. This is important as, like in other internet- and mobile-based interventions, safety management is difficult to perform, and we have tried to combat this by preparing a multilevel anti-suicidality approach. Another advantage is that participants in SOT will already receive some help that might lead to improvement, allowing unspecific working mechanisms to be controlled for. While we cannot assess long-term stability with the current design with a follow-up period of 4 weeks, the aim of the study is to assess, for the first time, the short-term efficacy in youth with BDD and to allow participants in the SOT condition to cross over to the ImaginYouth condition swiftly. Furthermore, we decided in favor of non-standardized messages after each session of ImaginYouth (and during the SOT) by the therapist, with the therapist instead adapting her comments to the patients. While this might limit generalizability, it might on the other hand decrease dropouts. And lastly, video but not audio recording during the diagnostic sessions will be non-mandatory, as this might hinder potential patients from participating. Thus, we cannot use this option to completely rule out any objective facial flaws in appearance (if other flaws are primary video recording would not be helpful anyway).

## Conclusion

To conclude, this internet-based therapist-guided CBT intervention is a treatment that aims to reduce symptoms in adolescents with BDD. Given the scarcity of intervention research in adolescents with BDD, this study will start to close this research gap, illustrating the efficacy of CBT and superiority over supportive therapy in adolescents with BDD, as well as providing information on the acceptability and feasibility of the online format. This format might be especially ideal when targeting this population, as it can be used independently of place and time, by affected individuals who are highly tech-savvy. Furthermore, it might make treatment for BDD more accessible despite high experiences of shame in youth with BDD, and in view of the current limited availability of high-quality treatment as well as long waiting lists.

## Author Contributions

AH, TS, DE, AM, and KS designed the study. AH and MS drafted the manuscript, supervised by KS. TS, DE, and AM contributed to the further writing of the manuscript. All authors read and approved the final manuscript.

## Conflict of Interest

The authors declare that the research was conducted in the absence of any commercial or financial relationships that could be construed as a potential conflict of interest.
